# Supplementary material for: Physical activity levels, exercise intrinsic motivation, physical fitness, and their association with adiposity and Oxytocin Receptor (OXTR) rs53576 and rs2254298 gene variants
Source: PLoS One. 2025 Sep 16;20(9):e0332672. doi: 10.1371/journal.pone.0332672 (PMC12440189; doi:10.1371/journal.pone.0332672)
Supplement: S1 Table — (DOCX) [file pone.0332672.s001.docx]

**Supporting Information**

**S1 Table. Association of OXTR rs53576 and rs2254298 alleles with demographics, PA level, intrinsic exercise motivation, physical fitness and anthropometric and body composition classes.**

|  | **rs53576 Alleles** | | **rs2254298 Alleles** | |
| --- | --- | --- | --- | --- |
|  | **A (*n* = 352)** | **G (*n* = 194)** | **G (*n* = 302)** | **A (*n* = 244)** |
| **Gender** |  |  |  |  |
| Male | 155 (65.7) | 81 (34.3) | 128 (54.2) | 108 (45.8) |
| Female | 197 (63.5) | 113 (36.5) | 174 (56.1) | 136 (43.9) |
| ꭓ^2^; *p* | 0.265; 0.607 | | 0.194; 0.660 | |
| **Ethnicity** |  |  |  |  |
| Malay/Bumiputra | 15 (46.9) | 17 (53.1) | 16 (50.0) | 16 (50.0) |
| Chinese | 315 (68.8) | 143 (31.2) | 245 (53.5) | 213 (46.5) |
| Indian | 22 (39.3) | 34 (60.7) | 41 (73.2) | 15 (26.8) |
| ꭓ^2^; *p* | 23.540; <0.001** | | 8.239; 0.016* | |
| **Physical Activity Category** |  |  |  |  |
| Low (<600) | 41 (58.6) | 29 (41.4) | 39 (55.7) | 31 (44.3) |
| Moderate (600-2999.99) | 164 (70.7) | 68 (29.3) | 132 (56.9) | 100 (43.1) |
| High (≥3000) | 147 (60.2) | 97 (39.8) | 131 (53.7) | 113 (46.3) |
| ꭓ^2^; *p* | 6.882; 0.032* | | 0.500; 0.779 | |
| **MPAM Interest Category** |  |  |  |  |
| Low | 81 (64.3) | 45 (35.7) | 64 (50.8) | 62 (49.2) |
| Medium | 176 (64.7) | 96 (35.3) | 159 (58.5) | 113 (41.5) |
| High | 95 (64.2) | 53 (35.8) | 79 (53.4) | 69 (46.6) |
| ꭓ^2^; *p* | 0.014; 0.993 | | 2.352; 0.308 | |
| **MPAM Competence Category** |  |  |  |  |
| Low | 72 (61.0) | 46 (39.0) | 64 (54.2) | 54 (45.8) |
| Medium | 205 (68.3) | 95 (31.7) | 167 (55.7) | 133 (44.3) |
| High | 75 (58.6) | 53 (41.4) | 71 (55.5) | 57 (44.5) |
| ꭓ^2^; *p* | 4.498; 0.105 | | 0.072; 0.965 | |
| **MPAM Appearance Category** |  |  |  |  |
| Low | 87 (62.1) | 53 (37.9) | 78 (55.7) | 62 (44.3) |
| Medium | 183 (70.4) | 77 (29.6) | 144 (55.4) | 116 (44.6) |
| High | 82 (56.2) | 64 (43.8) | 80 (54.8) | 66 (45.2) |
| ꭓ^2^; *p* | 8.698; 0.013* | | 0.026; 0.987 | |
| **MPAM Fitness Category** |  |  |  |  |
| Low | 87 (64.0) | 49 (36.0) | 77 (56.6) | 59 (43.4) |
| Medium | 181 (66.1) | 93 (33.9) | 146 (53.3) | 128 (46.7) |
| High | 84 (61.8) | 52 (38.2) | 79 (58.1) | 57 (41.9) |
| ꭓ^2^; *p* | 0.751; 0.687 | | 0.973; 0.615 | |
| **MPAM Social Category** |  |  |  |  |
| Low | 81 (65.3) | 43 (34.7) | 75 (60.5) | 49 (39.5) |
| Medium | 190 (63.8) | 108 (36.2) | 156 (52.3) | 142 (47.7) |
| High | 81 (65.3) | 43 (34.7) | 71 (57.3) | 53 (42.7) |
| ꭓ^2^; *p* | 0.145; 0.930 | | 2.590; 0.274 | |
| **Physical Fitness Category** |  |  |  |  |
| Good | 35 (60.3) | 23 (39.7) | 35 (60.3) | 23 (39.7) |
| Average | 37 (71.2) | 15 (28.8) | 26 (50.0) | 26 (50.0) |
| Poor | 280 (64.2) | 156 (35.8) | 241 (55.3) | 195 (44.7) |
| ꭓ^2^; *p* | 1.457; 0.483 | | 1.188; 0.552 | |
| **Blood Pressure Category** |  |  |  |  |
| Normal | 282 (63.5) | 162 (36.5) | 240 (54.1) | 204 (45.9) |
| Prehypertension | 62 (67.4) | 30 (32.6) | 56 (60.9) | 36 (39.1) |
| Stage 1 hypertension | 6 (75.0) | 2 (25.0) | 4 (50.0) | 4 (50.0) |
| Stage 2 hypertension | 2 (100) | 0 | 2 (100) | 0 |
| ꭓ^2^; *p* | 2.010; 0.570 | | 3.141; 0.370 | |
| **WC Class** |  |  |  |  |
| Normal | 324 (64.0) | 182 (36.0) | 280 (55.3) | 226 (44.7) |
| High | 28 (70.0) | 12 (30.0) | 22 (55.0) | 18 (45.0) |
| ꭓ^2^; *p* | 0.576; 0.448 | | 0.002; 0.967 | |
| **WHR Class** |  |  |  |  |
| Normal | 336 (64.9) | 182 (35.1) | 287 (55.4) | 231 (44.6) |
| High | 16 (57.1) | 12 (42.9) | 15 (53.6) | 13 (46.4) |
| ꭓ^2^; *p* | 0.692; 0.406 | | 0.036; 0.849 | |
| **WHtR Class** |  |  |  |  |
| Normal | 315 (64.3) | 175 (35.7) | 269 (54.9) | 221 (45.1) |
| High | 37 (66.1) | 19 (33.9) | 33 (58.9) | 23 (41.1) |
| ꭓ^2^; *p* | 0.070; 0.791 | | 0.330; 0.565 | |
| **TBF Class** |  |  |  |  |
| Normal | 245 (66.9) | 121 (33.1) | 196 (53.6) | 170 (46.4) |
| High | 107 (59.4) | 73 (40.6) | 106 (58.9) | 74 (41.1) |
| ꭓ^2^; *p* | 2.959; 0.085 | | 1.390; 0.238 | |
| **VFL Class** |  |  |  |  |
| Normal | 329 (64.3) | 183 (35.7) | 283 (55.3) | 229 (44.7) (33.2) |
| High | 23 (67.6) | 11 (32.4) | 19 (55.9) | 15 (44.1) |
| ꭓ^2^; *p* | 0.160; 0.689 | | 0.005; 0.945 | |
| **BMI Overweight Class** |  |  |  |  |
| Normal | 269 (65.0) | 145 (35.0) | 226 (54.6) | 188 (45.4) |
| Overweight | 83 (62.9) | 49 (37.1) | 76 (57.6) | 56 (42.4) |
| ꭓ^2^; *p* | 0.192; 0.661 | | 0.361; 0.548 | |
| **BMI Obese Class** |  |  |  |  |
| Normal | 332 (64.1) | 186 (35.9) | 288 (55.6) | 230 (44.4) |
| Obese | 20 (71.4) | 8 (28.6) | 14 (50.0) | 14 (50.0) |
| ꭓ^2^; *p* | 0.624; 0.430 | | 0.337; 0.562 | |
| **SM Class** |  |  |  |  |
| Normal | 218 (63.4) | 126 (36.6) | 201 (58.4) | 143 (41.6) |
| High | 134 (66.3) | 68 (33.7) | 101 (50.0) | 101 (50.0) |
| ꭓ^2^; *p* | 0.488; 0.485 | | 3.659; 0.056 | |

Parentheses indicate percentages out of the same demographic, PA level, intrinsic exercise motivation, physical fitness and anthropometric/body composition class.

BMI: Body Mass Index; TBF: Total Body Fat; VFL: Visceral Fat Level; SM: Skeletal Muscle Percentage; WC: Waist Circumference; WHR: Waist-Hip Ratio; WHtR: Waist-Height Ratio; MPAM: Motives for Physical Activities Measure

**p*-value is significant at the 0.05 level (2-tailed); ***p*-value is significant at the 0.01 level (2-tailed).
