# Supplementary material for: Physical activity levels, exercise intrinsic motivation, physical fitness, and their association with adiposity and Oxytocin Receptor (OXTR) rs53576 and rs2254298 gene variants
Source: PLoS One. 2025 Sep 16;20(9):e0332672. doi: 10.1371/journal.pone.0332672 (PMC12440189; doi:10.1371/journal.pone.0332672)
Supplement: Attachment — Submitted filename: Response to Reviewers OXTR.docx [file pone.0332672.s003.docx]

**Physical activity levels, exercise intrinsic motivation, physical fitness, and their association with adiposity and Oxytocin Receptor (OXTR) rs53576 and rs2254298 gene variants among Malaysian urban young adults**

**PONE-D-25-11325**

***PLOS One***

Dear Academic Editor,

We sincerely thank you and the reviewers for carefully reading our manuscript and providing constructive comments, and we have addressed them point-by-point and amended the manuscript, as follows:

| **Comments** | **Response to Reviewers** |
| --- | --- |
| **Editor** | |
| - Please ensure that your manuscript meets PLOS ONE's style requirements, including those for file naming. | - Manuscript amended to fulfil PLOS ONE’s style requirements. |
| - Your ethics statement should only appear in the Methods section of your manuscript. If your ethics statement is written in any section besides the Methods, please delete it from any other section. | - Ethical statement deleted from declarations section. |
| - In the online submission form, you indicated that data will be made available on request.   All PLOS journals now require all data underlying the findings described in their manuscript to be freely available to other researchers, either a. In a public repository, b. Within the manuscript itself, or c. Uploaded as supplementary information. | - Data availability amended to “All relevant data are within the manuscript.” |
| - We note that you have included the phrase “data not shown” in your manuscript. Unfortunately, this does not meet our data sharing requirements. PLOS does not permit references to inaccessible data. We require that authors provide all relevant data within the paper, Supporting Information files, or in an acceptable, public repository. Please add a citation to support this phrase or upload the data that corresponds with these findings to a stable repository (such as Figshare or Dryad) and provide and URLs, DOIs, or accession numbers that may be used to access these data. Or, if the data are not a core part of the research being presented in your study, we ask that you remove the phrase that refers to these data. | - Phrases “data not shown” removed. Additional data has been added as Supporting Information S1 Table. |
| - We note that you have provided funding information that is not currently declared in your Funding Statement. However, funding information should not appear in the Acknowledgments section or other areas of your manuscript. We will only publish funding information present in the Funding Statement section of the online submission form.   Please remove any funding-related text from the manuscript and let us know how you would like to update your Funding Statement. | - Funding statement removed from Acknowledgements. Declaration of Funding Statement in the online submission form has been amended to “This research is supported by the Malaysian Ministry of Higher Education Fundamental Grant Research Scheme FRGS/1/2022/STG01/SYUC/02/1. The funder had no role in the design of the study; in the collection, analyses, or interpretation of data; in the writing of the manuscript; or in the decision to publish the results.” |
| **Reviewer 1** | |
| - The authors are encouraged to perform interaction analyses between the investigated variables and the identified SNPs, in order to more robustly assess the strength and nature of these associations. | Although Structural Equation Modelling (SEM) is a powerful technique for examining complex, multi-pathway relationships among latent and observed variables, it was not employed in this study due to both methodological and practical considerations. First, the primary aim of this study was to examine direct associations and group differences among measured variables such as PA levels, intrinsic motivation subscales, physical fitness, adiposity, and OXTR genotypes. These objectives were more appropriately addressed using conventional statistical methods such as correlation, regression, and group comparisons.  Second, SEM requires large sample sizes to yield stable and valid estimates, particularly when models include multiple latent constructs and pathways. Given the modest sample size of this study and the inclusion of genetic data with categorical variants, SEM would have lacked the statistical power necessary to produce reliable model fit and parameter estimates. Moreover, this study focused on exploratory rather than confirmatory modelling, making simpler, theory-informed analyses more suitable at this stage.  Future studies with larger sample sizes and longitudinal designs may benefit from the use of SEM to better model mediating or moderating pathways between genetic, psychological, and behavioral variables.  The last point above has also been added to the limitations of study/future studies paragraph in Discussion. |
| - Although the manuscript states that the SNPs in question do not alter oxytocin protein levels, it does not provide mechanistic insight into how these polymorphisms might impact downstream signaling pathways. It is recommended that the authors incorporate in silico or in vitro analyses to investigate potential structural alterations in the oxytocin receptor and their functional implications for signal transduction. | In silico prediction of the effect of SNPs like SIFT, PolyPhen2, SNAp, PhD-SNP all are irrelevant, as the SNPs are not missense (protein-coding) SNPs.  SNPinfo search (<https://snpinfo.niehs.nih.gov/cgi-bin/snpinfo/snpfunc.cgi>) revealed no results for the SNPs.  A search in the VarSome database for rs53576 and rs2254298 (https://varsome.com/variant/hg19/rs53576? and https://varsome.com/variant/hg19/rs2254298?, respectively) revealed Deleterious Annotation of genetic variants using Neural Networks (DANN) scores of 0.6058 and 0.3451, indicating benign moderate and benign strong damaging effects of the SNP, respectively.  These SNPs are located in the intron containing OCE7, a cis-regulatory element (cCRE) that has a robust enhancer activity for *Oxtr* gene in mouse hypothalamus cells (Laboy et al., 2025), suggesting potential effects on behavioural phenotypes in humans. |
| - Furthermore, the employment of genetically engineered experimental models bearing the same or analogous mutations in the OXTR gene would offer a valuable opportunity to explore the molecular mechanisms by which such polymorphisms influence motivation and behavioral responses to physical exercise. | These SNPs are located in the intron containing OCE7, a cis-regulatory element (cCRE) that has a robust enhancer activity for *Oxtr* gene in mouse hypothalamus cells (Laboy et al., 2025), suggesting potential effects on behavioural phenotypes in humans. |
| - Lastly, it is suggested that the title of the manuscript be revised to reduce its regional specificity and to more effectively underscore the broader relevance of the observed associations between genetic polymorphisms and the phenotypic traits under investigation. | Title amended to “Physical activity levels, exercise intrinsic motivation, physical fitness, and their association with adiposity and Oxytocin Receptor (*OXTR*) rs53576 and rs2254298 gene variants” |
| **Reviewer 2** | |
| - Lack of Theoretical Integration:   The manuscript reads like multiple unrelated studies combined into one: a PA/motivation study, a fitness/adiposity study, and a genetics study. The relationships between these variables are not clearly explained, and no cohesive theoretical model is presented to justify their joint analysis. The importance and applicability of studying these variables together remain unclear. | Introduction has been rewritten to show interrelatedness between the multiple components of PA/motivation study, a fitness/adiposity study, and a genetics study. |
| - Overly Long and Unfocused Introduction:   The introduction contains excessive background information on common knowledge (e.g., definitions of motivation types). It lacks coherence, jumping from topic to topic without clearly connecting them. Although gaps are mentioned (e.g., lack of studies on motivation and PA, inconsistent gender findings, absence of Malaysian data), their significance is not convincingly justified. | Introduction has been rewritten to be shortened, more focused, and with the significance of study more justified. |
| - Redundancy in Methodology:   The methods section includes obvious and unnecessary statements, such as: “Participants who did not fulfil the inclusion criteria were excluded from the study.” Such redundancy detracts from the clarity and professionalism of the methods. | The sentence “Participants who did not fulfil the inclusion criteria were excluded from the study” is deleted. |
| - Insufficient Power for Genetic Analyses:   No power calculation is provided for the genetic component. Given the modest sample size (N=273), the study may be underpowered to detect meaningful genetic associations. | Using the Genetic Association Study Power Calculator (https://csg.sph.umich.edu/abecasis/cats/gas_power_calculator/), the statistical power for rs53576 and rs2254298 SNPs were 80% and 50%, respectively, assuming that overweight is the “disease” or outcome; the case/control sample size of 207 normal and 66 overweight; the significance level of 0.05; the disease model is additive; the prevalence of overweight among Malaysians is 31.3% [24]; the disease allele frequency is 0.36 and 0.45 for rs53576 and rs2254298, respectively; and the heterozygous genotype relative risk is 1.532 and 1.316, respectively Due to the weak statistical power particularly for rs2254298, this limitation has been added to the Discussion. |
| - Weak Justification for Genetic Focus:   The study claims to be the first to assess OXTR variants with PA and motivation, but the biological mechanism linking these variables is unclear. Prior associations of OXTR are mostly in the context of social, emotional, or psychiatric traits, not physical activity behavior. | A stronger justification has been provided in Discussion as follows:  Although OXTR gene variants have primarily been studied in relation to social and emotional traits [9], their influence on PA is biologically plausible. Oxytocin interacts with the brain’s reward and motivation systems, particularly the dopaminergic pathway, which may affect how individuals experience intrinsic rewards from PA [14]. Since intrinsic motivation is shaped by enjoyment, competence, and social connection—domains where oxytocin is active—OXTR gene variants may help explain individual differences in PA behaviour. While this genetic link remains underexplored, especially in non-Western populations, examining OXTR in relation to PA and motivation offers a novel, biologically grounded perspective to understanding PA engagement. |
| - Use of Self-Reported PA Measures:   PA is assessed using the self-reported IPAQ-SF, which is prone to recall and social desirability bias. Drawing associations between self-reported PA and biological/genetic variables is questionable without objective PA data (e.g., accelerometry). | This has already been mentioned in the list of limitations of the study as follows:  To complement self-reported measures, accelerometer or activity tracker bands could be used to objectively track physical activities. |
| - Missing Methodological References:   No reference is provided for the genotyping protocol used. No citation is given for the interpretation of correlation coefficient thresholds either (e.g., what defines weak, moderate, strong). | Genotyping was performed according to the manufacturer’s protocols. This has been added to the Methods:  Genomic DNA was extracted from mouthwash samples using the GF-1 Tissue DNA Extraction Kit (Vivantis, Malaysia), before proceeding for genotyping of OXTR rs53576 and rs2254298 using the FRET (fluorescent resonance energy transfer) chemistry allele-specific real-time PCR-based KASP™ genotyping assay (LGC Biosearch Technologies, UK) according to the manufacturer’s protocols.  A reference for the interpretation of the correlation values has been provided: Evans J. Straightforward statistics for the behavioral sciences. Brooks/Cole Publishing; Pacific Grove, California; 1996. |
| - Causal Language in Discussion of Cross-Sectional Data:   Although the authors note the study is cross-sectional, the discussion includes causal statements (e.g., “intrinsic motivation leads to more PA, which reduces adiposity”), which are inappropriate for the study design. | Causal language removed. Sentence changed to:  Higher Interest and Competence were correlated with higher engagement hours in vigorous, moderate, and total PA, but were correlated with lower engagement hours in mundane physical activity (walking) and sedentary activity (sitting). |
| - Ambiguity in Socioeconomic Classification:   The term “M40” is used without explanation. What does it stand for? | The definitions of B40, M40, and T20 are already given in the footnote of Table 1.  An additional definition of M40 has also been added to paragraph 1 of Results:  …M40 monthly household income category (considered as middle-income group with the monthly income of MYR 4851-10,960)… |

With the above amendments, it is our great hope that our manuscript will be accepted for publication. Thank you.
